# Indole-3-acetic acid as a cross-talking molecule in algal-bacterial interactions and a potential driving force in algal bloom formation

**DOI:** 10.3389/fmicb.2023.1236925

**Published:** 2023-10-20

**Authors:** Xueyu Cheng, Xinyang Li, Mengmeng Tong, Jiajun Wu, Leo Lai Chan, Zhonghua Cai, Jin Zhou

**Affiliations:** ^1^Shenzhen Public Platform for Screening and Application of Marine Microbial Resources, Institute for Ocean Engineering, Shenzhen International Graduate School, Tsinghua University, Shenzhen, China; ^2^The Direction of Deep Sea Resource Exploration and Development Utilization, Hainan Institute of Zhejiang University, Sanya, China; ^3^State Key Laboratory of Marine Pollution, City University of Hong Kong, Kowloon, Hong Kong SAR, China

**Keywords:** indole-3-acetic acid, signaling, signaling molecule, cross-kingdom communication, algal-bacterial interaction, algal bloom

## Abstract

Most signaling molecules are involved in inter-or intra-species communication, and signaling involving cross-kingdom cell-to-cell communication is limited. Howerver, algae and bacteria exchange nutrients and information in a range of interactions in marine environments. Multiple signaling molecules exist between algae and bacteria, including quorum-sensing molecules, nitric oxide, and volatile organic compounds. Recently, indole-3-acetic acid (IAA), an auxin hormone that is a well-studied signaling molecule in terrestrial ecosystems, was found to act as a cue in cross-kingdom communication between algae and bacteria in aquatic environments. To increase understanding of the roles of IAA in the phycosphere, the latest evidence regarding the ecological functions of IAA in cross-kingdom communication between algae and bacteria has been compiled in this review. The pathways of IAA biosynthesis, effects of IAA on algal growth & reproduction, and potential mechanisms at phenotypic and molecular levels are summarized. It is proposed that IAA is an important molecule regulating algal–bacterial interactions and acts as an invisible driving force in the formation of algal blooms.

## 1. Introduction

Interactions between plants and symbiotic bacteria in rhizosphere microenvironments have been extensively studied in terrestrial ecosystems (Philippot et al., 2013). Similar to the terrestrial rhizosphere, marine environments have the “phycosphere” associated with algae (Cole, 1982). In the phycosphere niche, interactions between algae and bacteria are highly complex and diverse, ranging from cooperation to competition (Amin et al., 2015). Recently, on account of the high structural and functional diversity observed, the complex interactions between algae and bacteria have become the focus of research (Ramanan et al., 2016; Brodie et al., 2017; Piampiano et al., 2020). Algal–bacterial relationships are mediated by various factors, including biological parameters, environmental conditions, and chemical cues, which depend on population and community structures. Most recently, chemical cues have received particular attention (Hay, 2009).

Molecular cross-talk between bacteria and eukaryotes has been described in a wide range of symbiotic organisms (Pacheco and Sperandio, 2009), involving chemical signals such as quorum-sensing substances, hormones, and small-molecule compounds. Studies of cross-kingdom communication can broaden the understanding of multi-species interactions (Sandhya and Vijayan, 2019; Piampiano et al., 2020; Wang et al., 2022). However, compared with studies of inter-and intra-species communication, research examining eukaryotic–bacterial interactions mediated by cross-kingdom signals is limited. Indole-3-acetic acid (IAA) is a plant hormone belonging to the auxin class and is the cross-kingdom signal known to mediate plant–bacteria relationships. Bacteria develop colonization strategies in plant–microorganism interactions by producing IAA, including phytostimulation and circumvention of basal plant defense mechanisms (Duca et al., 2014; Kunkel and Harper, 2017). Furthermore, IAA is also a signaling molecule in bacteria and can directly affect bacterial physiology (Spaepen et al., 2007; Fu et al., 2015).

Although the roles of IAA in terrestrial environments have been investigated, little is known about the functions of IAA in interactions between algae and associated microbes in aquatic environments (Lin et al., 2022). Signaling molecules are important in regulating algal symbioses, including nutrient cycling, material transport, symbiotic interactions, and coevolution, which in turn mediate the relationship between algae and bacteria, thereby affecting algal life cycles and the demise of algal blooms (Labeeuw et al., 2016; Zhou et al., 2016; Barak-Gavish et al., 2023). This review focuses on recent studies elucidating IAA signals and their ecological functions in symbiotic interactions between algae and bacteria. The objectives were to identify the major mechanisms driving the establishment of algal–bacterial interactions and to determine the potential mechanisms driving the development of algal blooms. Such a synthesis of new information is essential to integrate current knowledge about how inter-kingdom cross-talk affects algae–bacteria relations and the mechanisms of algal bloom formation.

## 2. Algal–bacterial interactions in the phycosphere

The phycosphere niche provides a rich environment for heterotrophic bacteria in contrast to the poor trophic environment in open marine systems, thus serving as a bacterial “oasis” (Zhang et al., 2018). Within the phycosphere microenvironment, algae release fixed organic carbon and maintain high nutrient concentrations to attract and support the growth of heterotrophic bacteria (Azam and Malfatti, 2007). In return, bacteria provide vitamins and trace elements that algae cannot biosynthesize, including vitamins B_1_ and B_12_ and iron, which are necessary cofactors for the biosynthesis of various enzymes by algae (Ramanan et al., 2016).

Mutualistic relationships between vitamin-synthesizing bacteria and algae that require exogenous vitamin inputs have been studied extensively. Croft et al. (2005) analyzed the vitamin sources of 326 algal species and discovered that 171 species required exogenous vitamin B_12_ to biosynthesize methionine. They also demonstrated that a bacterium from the genus *Halomonas* increased production of vitamin B_12_ when provided with fucoidin, a commercial algal extract, which they interpreted as evidence that bacteria supply vitamins (particularly vitamin B_12_) on a global scale to most vitamin B_12_-auxotrophic phytoplankton in exchange for fixed carbon. Algal–bacterial co-culture experiments further demonstrate that algae acquire vitamin B_12_ by interacting with bacteria. The medium of the co-cultures did not contain an organic carbon source, and therefore the bacteria were presumably using the products of algal photosynthesis to grow, suggesting that this is a mutualistic relationship (Croft et al., 2005). Moreover, exchanges of iron and carbon between several *Marinobacter* species and phytoplankton, such as diatoms, dinoflagellates, and coccoliths, are indicative of algal–bacterial mutualism (Amin et al., 2009). Bacteria produce vibrioferrin, a siderophore that binds to trace amounts of iron in the open ocean, facilitating algal assimilation of iron by enabling photochemical redox cycling of this vital nutrient; simultaneously, algae release organic molecules that support bacterial growth (Amin et al., 2009). There is an exchange of ammonium and diatom-secreted taurine (a carbon source) between *Sulfitobacter* and *Pseudonitzschia multiseries* (Seymour et al., 2017). De-Bashan et al. (2008) demonstrated the transfer of carbon and nitrogen compounds between *Chlorella sorokiniana* and *Azospirillum brasilense*, an interaction that benefits both partners and can remain stable over multiple generations. Mutualism is not limited to unicellular microalgae but is also prevalent in macroalgae and, in some cases, the mutualistic relationship is endosymbiotic (Hollants et al., 2011).

Ecological relations between algae and bacteria can be classified as not only mutualism but also as competitive antagonism (Ramanan et al., 2016; Seymour et al., 2017). To compete for limited material, such as inorganic nutrients, bacteria can secrete algaecides to kill algae and algae can defend themselves against bacterial attacks. For example, the flavobacterium *Kordia algicida* releases a protease, with a mass of >30 kDa, that fatally targets a subset of diatoms (*Skeletonema*, *Thalassiosira*, and *Phaeodactylum*, but not *Chaetoceros*; Amin et al., 2012). The marine isolate, *Saprospira* sp. strain SS98-5, lyses cells of the diatom *Chaetoceros ceratosporum* on direct contact. The bacterium uses gliding motility to swim toward the diatom and induces diatom cell aggregation, followed by lysis *via* the production of microtubule-like structures (Furusawa et al., 2003). Abada et al. (2023) reported that, under oxygen-rich conditions, the aerobic bacterium, *Phaeobacter inhibens*, reduced algal-secreted nitrite to nitric oxide (NO) through denitrification. The bacterial NO is involved in triggering a cascade of reactions in the coccolithophore *Gephyrocapsa huxleyi* algae that is akin to programmed cell death. Upon death, algae generate additional NO, thereby propagating the signal within the algal population. Eventually, the algal population collapses, similar to the sudden demise observed in oceanic algal blooms. In turn, the diatoms possess defense mechanisms to protect themselves. The diatom *Navicula delognei* produces several antibacterial compounds; three of these compounds – the fatty acids hexadecatetraenoic acid, octadecatetraenoic acid, and the ester (*E*)-phytol – display strong antibacterial activity against the pathogens *Staphylococcus aureus, Staphylococcus epidermidis, Proteus vulgaris,* and *Salmonella enterica* serovar Typhimurium (Findlay and Patil, 1984). *Phaeodactylum tricornutum* can produce the fatty acids palmitoleic acid and eicosapentaenoic acid (EPA), both of which inhibit the growth of Gram-positive bacteria (Desbois et al., 2008).

In addition to the sharing of nutrition, space, and material exchange, the relationship between an algal host and epiphytic bacteria can be also regulated by signaling molecules. For example, dimethylsulfonylpropionate (DMSP) released by algae can stimulate the pathogenicity of bacteria, changing the relation between *Sulfitobacter* D7 and *Gephyrocapsa huxleyi* from “good friends” to “enemies” (Barak-Gavish et al., 2023). During rapid growth of an *G. huxleyi* population, organic matter is released into the phycosphere and *Roseobacter* D7 attaches to algal cell walls to efficiently use the nutrients released by the algae. Simultaneously, the bacterium provides vitamins and plant hormones to the alga, thus strengthening the “friendship.” However, as algal physiology deteriorates and partial death begins, the concentration of algae-derived DMSP begins to increase (Sule and Belas, 2012). When the change in DMSP concentration is detected, bacteria detect that the algal biochemical state is degenerating and, consequently, begin to regulate the expression of pathogenic-related genes and release algicidal substances. Thus, the bacteria take advantage of “old friends” by killing off algae hosts (Barak-Gavish et al., 2023).

Another group of important signaling molecules is the acyl-L-homoserine lactones (AHLs), which are self-inducing molecules that are commonly produced by bacteria to regulate bacterial behavior (Cuadrado-Silva et al., 2013). Moreover, AHLs participate in communication not only between bacteria but also between microbes and their algal hosts (Zhou et al., 2016). Two types of bacteria associated with diatoms can secrete different AHLs, which accumulate in diatom hosts (Amin et al., 2012) and cause their dissolution (Zhou et al., 2016). In response to bacterial AHL signals, algae have developed mechanisms to regulate their metabolism. For example, the red alga (macroalga) *Delisea pulchra* produces halogenated furanones, which inhibit the expression of AHL-dependent genes and, in turn, inhibit quorum sensing in bacteria. Algae have likely evolved in response to the negative impacts of AHL-dependent colonization of their surfaces by marine bacterial species (Maximilien et al., 1998; Zhu and Winans, 2001; Hughes and Sperandio, 2008). In a study, Amin et al. (2012) summarized that signaling molecules not only initiate or regulate algal–bacterial interactions concerning nutrient competition, but they also maintain partnerships that are more specific than those maintained solely *via* the exchange of nutrients. Considering the complexity of algal–bacterial interactions, it is not surprising that a large number of signaling molecules have been identified, such as indole-3-acetic acid (IAA).

## 3. What are the sources of indole-3-acetic acid (IAA) in the phycosphere?

### 3.1. Pathways of IAA biosynthesis in microalgae

In the 1960s and 1980s, IAA and its inactive analogs were discovered in various types of algae (Tarakhovskaya et al., 2007). In the 1990s, Stirk and Van Staden (1996) observed improved plant growth with the addition of small amounts of extracts from brown algae, similar to that induced by the plant growth hormone auxin. Endogenous IAA is found in a wide range of microalgal phyla, including 20 algal strains in the Haptophyta, Chlorophyta, and Streptophyta (Žižková et al., 2016). However, it cannot be concluded that an alga produces endogenous IAA based solely on the detection of IAA since detection alone cannot explain the large amounts of IAA found in algae without terrestrial IAA biosynthetic homologs (Taya et al., 2022). The metabolic pathways of auxin biosynthesis in microalgae are complex and have not been fully elucidated. Until now, the most likely putative IAA biosynthetic pathway in microalgae is via the oxidation of tryptophan (Trp) to indole-3-acetaldoximine (IAN; Lin et al., 2022). Characterization of the genes and proteins responsible for IAA biosynthesis in microalgae lags far behind that in plants and bacteria.

**Figure 1:**
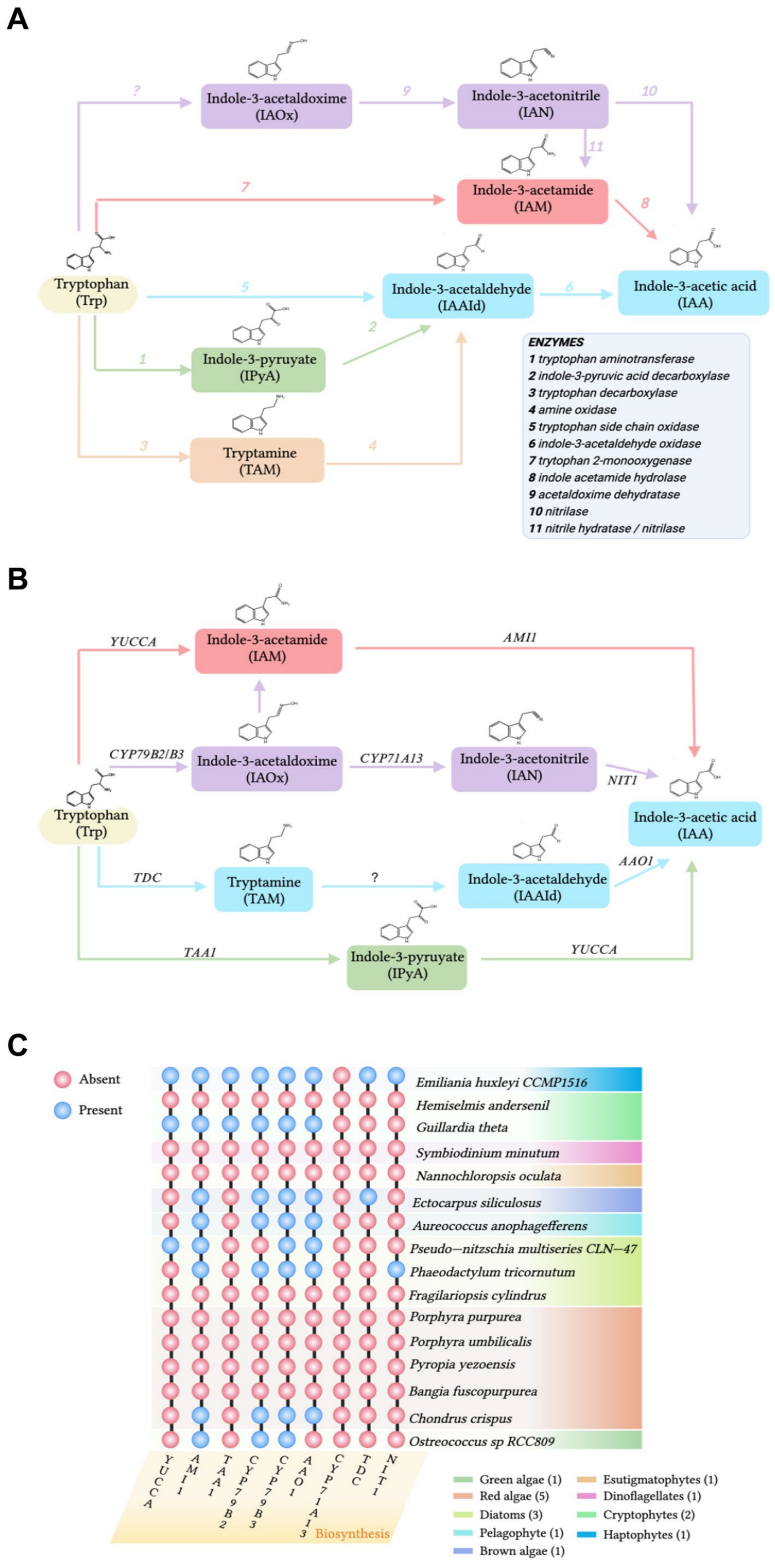
**(A)** The pathways of biosynthesis of indole-3-acetic acid (IAA) in bacteria. Enzymes are labeled by number in the legend. **(B)** The tryptophan (Trp)-dependent IAA biosynthesis pathways in *A. thaliana*. AAO1: indole-3-acetaldehyde oxidase; AMI1: indole-3-acetamide hydrolase; CYP79B2 and its close homolog CYP79B3: cytochromes that catalyze the conversion of Trp to indole-3-acetaldoxime; CYP7A13, indole-3-acetaldoxime dehydratase; NIT1, Nitrilase; TAA1, tryptophan aminotransferase; TDC, tryptophan decarboxylase; and YUCCA: flavin-containing monooxygenases. **(C)** Distribution of genes involved in IAA biosynthesis from the terrestrial plant *Arabidopsis thaliana* detected in the genomes of various algal species. Abbreviations of enzymes involved in the Trp-dependent IAA biosynthesis pathways of *A. thaliana* are defined in **(B)**. Orthologs present (blue circles) or absent (pink circles). Figure based on findings from Mashiguchi et al. (2011), Labeeuw et al. (2016), Mikami et al. (2015), and Carrillo-Carrasco et al. (2023).

To explore the sources of IAA in the phycosphere, both the accumulation of IAA in algae and epiphytic bacteria and the gene homologs of the IAA biosynthetic pathway need to be examined. Thus, chemical and molecular evidence need to be combined to explore the sources of IAA in the phycosphere. The IAA biosynthetic pathways of algae and *Arabidopsis thaliana* have been compared to investigate if there are homologs of *A. thaliana* biosynthetic genes in the algae (Figures 1B,C). In a comparison of the genomes of *A. thaliana* and multiple marine algae IAA biosynthetic pathways, Labeeuw et al. (2016) collected sequences for enzymes in the Trp-dependent IAA biosynthesis pathways of *A. thaliana* and queried homologs in algae databases (Figures 1B,C). Mikami et al. (2015) analyzed genes involved in IAA biosynthesis in red algae by searching the whole genomes of the red seaweeds *Bangia fuscopurea* and *Pyropia yezoensis*. The overview of the distribution of IAA biosynthetic genes in the algal genome is given (Figure 1C). Although some IAA-containing algae have homologs of genes from the *A. thaliana* Trp-dependent IAA biosynthetic pathway (Labeeuw et al., 2016; Figure 1C), most marine algae do not have IAA biosynthetic gene homologs (Spaepen et al., 2007; Lin et al., 2022; Figure 1C). Consequently, the origin of most algal IAA is unclear. IAA may still be algal-produced, because even though IAA biosynthetic homologous enzymes or gene homologs are not detected, algae may have IAA biosynthetic pathways that are different from those of terrestrial plants (Spaepen et al., 2007). However, the statement that “algae follow a different IAA biosynthetic pathway than that of terrestrial plants” is not entirely accurate, because some algae do contain homologs of IAA biosynthetic genes from terrestrial plants (Le Bail et al., 2010; Carrillo-Carrasco et al., 2023; Figure 1C). Therefore, it is proposed that algae without IAA synthetic gene homologs accumulate IAA under the influence of phycosphere bacteria. Additionally, several compounds previously believed to be produced by eukaryotic hosts were later found to be produced by bacterial symbionts (Li and Vederas, 2009). To obtain additional supporting evidence, future studies should select appropriate model strains and characterize *in vitro* enzymes to assign biochemical classification to the products of IAA biosynthetic genes.

### 3.2. Pathways of IAA biosynthesis in microalgae-associated bacteria

Because IAA-producing bacteria are ubiquitous in the phycosphere and numerous epiphytic bacteria produce IAA, bacteria are the likely source of IAA (Wang et al., 2022). Four Trp-dependent pathways for IAA biosynthesis have been identified in bacteria: indole-3-acetamide (IAM), indole-3-pyruvate (IPyA), tryptamine (TAM), and indole-3-acetaldoxime (IAOx; Spaepen et al., 2007; Labeeuw et al., 2016; Duca and Glick, 2020; Figure 1A).

Two strains of epiphytic bacteria, *Alteromonas* sp. Mab 25 and *Labrenzia* sp. Mab 26, from the phycosphere of the marine microalga *Isochrysis galbana* S002, can produce IAA when Trp is present in the growth medium (Sandhya and Vijayan, 2019). Among 203 strains isolated from the phycosphere of the marine green algae *Tetraselmis suecica*, 30 strains belonging to 10 different genera were found to produce IAA (Piampiano et al., 2020). *Neptunomonas* sp. BPy-1 isolated from the marine red algae *P. yezoensis* and *Neptunomonas* sp. BZm-1, obtained from *Zostera marina*, can also produce IAA in a growth medium containing Trp (Matsuda et al., 2017). Although detection of IAA is generally straightforward, the process is time-consuming and laborious when many bacteria need to be examined. Therefore, when there are many bacterial samples, macroscopic verification is performed using metagenomic and metatranscriptomic. Wang et al. (2022) analyzed the microbiome of the *P. haitanensis* phycosphere and found that over 50% of the bacterial population contained genes from the four established IAA biosynthetic pathways (Figure 1A). Zhang et al. (2019) analyzed over 7,000 bacterial genomes and discovered that 82.2% of genomes suggested that bacteria could potentially biosynthesize IAA from Trp or its intermediates. Thus, the use of experimental techniques to detect IAA and omics approaches to confirm IAA biosynthetic genes demonstrate that phycosphere bacteria can widely use Trp or its intermediates to generate IAA.

As for the ecological function of IAA, some researchers have pointed out that bacteria use IAA as a carbon or nitrogen source, either to increase adaptability to environmental pressures or to increase chemotaxis, which is beneficial for bacterial colonization (Lin et al., 2022). However, a consensus view recently emerged that IAA serves as a signaling molecule, participating in algal–bacterial interactions and influencing algal metabolism and growth.

## 4. The role of IAA as a signaling molecule affecting algal–bacterial relationships and regulating algal growth

**Figure 2:**
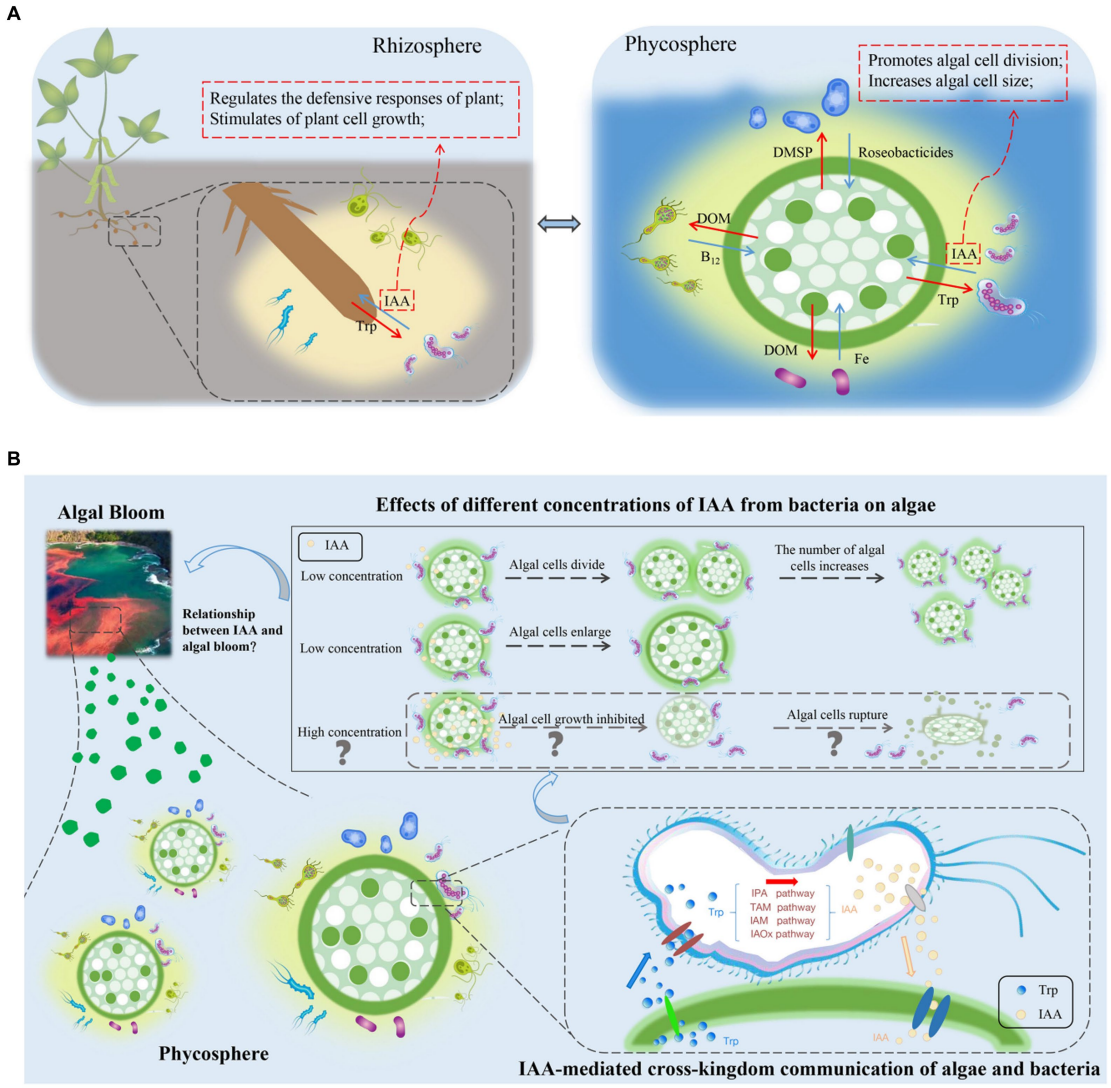
**(A)** Comparison of the rhizosphere and phycosphere environments and potential signaling roles of indole-3-acetic acid (IAA). DMSP, dimethylsulfonylpropionate; DOM, dissolved organic matter; IAA, indole-3-acetic acid; Roseobacticides, algaecides that kill algae; Trp, tryptophan. **(B)** Effects of IAA, produced by phycosphere bacteria, on the growth of algae, and a conceptual model of potential relationships between IAA-induced algal growth and algal blooms. IAA, indole-3-acetic acid; IAM, indole-3-acetamide; IAOx, indole-3-acetaldoxime; IPA, indole-3-pyruvate; TAM, tryptamine; Trp, tryptophan.

Within the rhizosphere, plant growth-promoting bacteria can biosynthesize IAA, which may be their most prominent characteristic (Cuadrado-Silva et al., 2013). In addition to promoting plant growth and development, IAA is also a signaling molecule involved in communication between plants and bacteria. In *A. thaliana*, the bacterial pathogen *Pseudomonas syringae* DC3000 impedes the salicylate-mediated defense capacity by secreting IAA signaling molecules, which consequently facilitate pathogen invasion (Djami-Tchatchou et al., 2020). In the phycosphere of a green alga, Durán et al. (2022) discovered several species of bacteria that produce IAA. Given the prevalence of IAA in the phycosphere and its role as a “cross-kingdom messenger” in the rhizosphere (Amin et al., 2015; Mori et al., 2017; Figure 2A), it is reasonable to speculate that IAA in the phycosphere also functions as a signaling molecule facilitating cross-kingdom communication between algae and bacteria, potentially affecting growth and population dynamics.

IAA produced by bacteria has important effects on macroalgal growth and morphogenesis. For example, overproduction of IAA by *Roseobacter* has long been known to cause localized galls in its algae host *Prionitis lanceolata* (Ashen et al., 1999). In the case of *Chondracanthus chamisoi*, the addition of exogenous IAA promotes the growth of intermediary nodes of its sterile explants and stimulates callus formation, likely because of its function during cell division and elongation (Yokoya et al., 2013). In addition, co-culturing *P. yezoensis* with IAA producers increases its growth (Matsuda et al., 2017).

In contrast to macroalgae, bacteria can manipulate the size and number of microalgae by providing exogenous IAA to influence growth, metabolism, and gene expression. De-Bashan et al. (2008) found that algal growth is promoted in the presence of wild-type *Azospirillum* spp., which produce varying amounts of IAA. In the relationship between the green microalga *Haematococcus pluvialis* and its symbiotic bacterium *Achromobacter* sp. CBA4603, the IAA-producing *Achromobacter* significantly increased algal growth after inoculation into *H. pluvialis* (Lee et al., 2019). Exogenously provided IAA also affects the size of algae. For example, when sterile *G. huxleyi* CCMP2090 is provided with exogenous IAA, algal morphology changes and cells enlarge significantly (Labeeuw et al., 2016). In addition to positive effects, there can also be negative feedback effects of IAA on algae. Therefore, IAA is a signaling molecule that can strengthen symbiotic relationships between algae and bacteria but, at high concentrations, it can also lead to algal death. Segev et al. (2016) reported that IAA at concentrations ranging from 1 to 100 μM had positive effects on algal growth, whereas concentrations exceeding 1,000 μM resulted in the death of 90% of algal cells. However, in natural marine environments, levels of IAA are not sufficient to cause death but most likely promote algal growth (Carrillo-Carrasco et al., 2023).

To determine whether IAA acts as a signaling molecule in algal–bacterial communication, it is not sufficient to demonstrate that bacterial-produced IAA influences the physiological state of algae and regulates algal gene expression, because IAA can also act as a nutrient for algae. Therefore, to provide additional compelling evidence supporting the role of IAA as a signaling molecule, it is necessary to demonstrate that IAA can selectively maintain algal–bacterial relationships, which is one of the most crucial characteristics of signaling molecules. Such specificity of the role of IAA can explain the different responses of the closely related *P. multiseries* (PC9、PC4、GGA2) and *Sulfitobacter* SA11 (Amin et al., 2015). For instance, in co-culture experiments, the presence of SA11 promoted cell division of *P. multiseries* PC9 but did not affect the other two algae, PC4 and GGA2. Furthermore, when PC9 and GGA2 were co-cultured with SA11, the number of SA11 cells increased, but the number of SA11 cells was not affected when co-cultured with PC4 (Amin et al., 2015). The observations indicate that IAA molecules help SA11 to distinguish between the strains of PC9, PC4, and GGA2 and establish contact with PC9 to promote cell reproduction in both the alga and bacterium. Therefore, IAA can help organisms differentiate between closely-related others and identify organisms with which it can interact. Under such circumstances, IAA facilitates the identification and maintenance of beneficial partnerships while relationships with deceivers are avoided; this resembles the specificity achieved by multiple signaling molecules in legume–rhizobia interactions (Wang et al., 2022).

The molecular mechanism by which bacteria-produced IAA regulates algal growth was studied in a co-culture of the diatom *Pseudo-nitzschia multiseries* and the bacterium *Sulfitobacte*r SA11 (Amin et al., 2015). Transcriptome sequencing and targeted metabolite analyses indicated that IAA was biosynthesized by SA11 using exogenous Trp and endogenous Trp secreted by *P. multiseries*. After IAA entered an algal cell, cell-cycle proteins in the nucleus detected the IAA and, in turn, promoted algal cell division. Three main functional genes responded to IAA. The first gene, encoding a protein involved in Trp biosynthesis and IAM-mediated IAA biosynthesis, was upregulated, leading to an increase in IAA that was taken up by the algae. The second gene is involved with cyclins, which are typically associated with growth promotion through the cell cycle. Expression of the third gene, the cysteine dioxygenase gene (*cdo*), also increased upon exposure to bacterial IAA and, as a result, taurine biosynthesis increased (Amin et al., 2015). Taurine is a critical carbon and energy resource for heterotrophic bacteria (Cook, 2006). Together with algae-derived DMSP, the release of taurine from algae may support the metabolism of algal-associated bacteria.

To explain why IAA can engage in cross-border communication between bacteria and hosts, partial answers can be obtained from terrestrial plants. IAA is a well-studied signaling molecule in terrestrial plants that participates in plant–microbial cross-kingdom communication. Several proteins are involved in IAA sensing and transport. Auxin-binding protein1 (ABP1) functions as an auxin receptor in the apoplast, whereas auxin transporters on the cell membrane, encoded by genes of the auxin resistant/like auxin resistant (AUX/LAX) family, transport extracellular IAA into cells. Pin-formed (PIN) proteins on the plasma membrane are responsible for IAA output, while PIN-like (PILS) transporters, localized to the endoplasmic reticulum, are responsible for IAA transport. ATP-binding cassette B (ABCB) transporters on the cell membrane act as IAA efflux carriers (Carrillo-Carrasco et al., 2023). When the concentration of IAA in the nucleus is high, IAA binds to the transport inhibitor response1-auxin signaling F-BOX proteins (TIR1/AFB family) and the auxin/IAA proteins (encoded by the Aux/IAA gene family), resulting in the degradation of Aux/IAA and release of the auxin response factor (ARF). Subsequently, ARF activates the transcription of IAA-reactive genes (Lau et al., 2009; Carrillo-Carrasco et al., 2023). The participation of numerous transporters ensures that IAA produced by bacteria affects the expression of endogenous IAA genes within plant cell nuclei.

It is important to note that the genes corresponding to the proteins responsible for sensing and transport of IAA in terrestrial plants are not currently known in algae. Although co-cultivation with IAA producers increases the growth of *P. yezoensis*, exogenous provision of IAA does not have the same effect. Genomic studies of *P. yezoensis* reveal the absence of gene homologs encoding auxin signaling components, such as the auxin efflux carrier (PIN) and auxin influx carrier (AUX1; Matsuda et al., 2017). This absence suggests that the mechanism of IAA transport in *Pyropia* is likely to differ from that in terrestrial plants and algae may absorb IAA directly from attached bacteria, rather than use IAA from the surrounding environment. Similarly, *P. yezoensis* and *P. umbilicalis*, which contain IAA *in vivo*, lack both homologs of IAA biosynthetic genes and proteins involved in IAA sensing and signal transduction. To further investigate the absence of genes homologs responsible for sensing and transport, the presence of TIR1/AFBs, Aux/IAA, and ARF orthologs in marine microalgae has been examined, but direct congeners of those proteins were not identified in *Ostreococcus lucimarinus*, *O. tauri* (Chlorophyta), *Phaeodactylum tricornutum*, *Thalassiosira pseudonana* (Bacillariophyta), and *Ectocarpus siliculosus* (Phaeophyta; Lau et al., 2009; Le Bail et al., 2010). Although many studies report that IAA acts as a signaling molecule and mediates cross-kingdom communication between algae and bacteria in marine ecosystems, the specific molecular mechanisms for this role remain uncertain.

## 5. IAA’s Potential role in algal blooms

Although algae are a key component in marine ecosystems, rapid proliferation of some algae can trigger algal blooms that can harm humans and other organisms (Berdalet et al., 2015). In forming an algal bloom, algae need to establish dominance, enabling them to increase faster than those of associated species (Huisman et al., 2018). A dominant advantage is a common feature of biological interactions between species, including algal–bacterial interactions. Regulation of chemical signals is important in algal–bacterial interactions (Jeong et al., 2015).

As a signaling molecule, IAA can regulate the competitive interactions between bacteria. Algal cells in the phycosphere not only secrete nutrients for bacteria but also provide nutrients in forms and concentrations that promote bacterial growth. Moreover, algal cells release signaling substances, such as Trp, that regulate bacterial gene expression. Bacteria respond to exogenous Trp signaling by altering their IAA production and converting all the Trp provided by the host alga into IAA and releasing it (Amin et al., 2015). The release of IAA leads to an increase in algal cell size and promotes cell division.

Algal cell size is the primary determinant of the size of the phycosphere (Seymour et al., 2017). The phycosphere extends from the surface of algal cells to the diffusion boundary layer that contains amino acids, carbohydrates, and other organic compounds released by algae (Seymour et al., 2017). In previous work, Wu et al. (2023) identified a positive feedback loop in the expansion of the phycosphere that resulted in bloom outbreaks. As the phycosphere expands, algae increase nutrient capture, and the semi-enclosed environment provides an exclusive nutrient supply for algal cells. Consequently, the amounts of nutrients available for bacteria increases, promoting the growth of IAA-producing bacterial populations. Such a positive feedback loop amplifies the strength of algae–bacterial interactions, leading to increases in algal populations and increased probability of algal blooms (Figure 2B). The implications of the positive feedback loop for large-scale biogeochemical processes are substantial. In studies that used omics techniques to analyze the entire cycle of algal blooms, IAA genes were found to be most highly expressed in the early stages of algal blooms, followed by expression in intermediate periods, with the lowest expression in the late stage (Zhu et al., 2022). This finding supports the hypothesis that algal blooms form because IAA promotes the growth and reproduction.

IAA-mediated interactions between algae and bacteria occur in specific microenvironments and can have cascading effects on primary productivity in the marine ecosystem and trigger algal blooms (Seymour et al., 2017). The secretion of IAA and its function as a signaling molecule in facilitating algal growth are potentially important in inducing algal bloom formation. Therefore, it is proposed that IAA acts as an invisible driving force in the development of algal blooms.

## 6. Outlook

The rapid progress of IAA research in the phycosphere niche is inspiring and will promote the study of IAA metabolism in algae as well as its role in algal blooms. Interactions between bacteria and algae, which involve exchanges of nutrients and information, are common in marine ecosystems. Bacteria detect Trp released by algae and, as a result, regulate the expression of IAA-associated genes and release IAA, which significantly affects algal growth and algal population size, by regulating cell size and number. Furthermore, IAA acts as a signaling molecule that maintains the partnership between algae and bacteria by transmitting information.

The discovery of the role of IAA in promoting algal growth and reproduction offers new insights into the development of algal blooms, yet the mechanisms remain unconfirmed. It also remains to be studied how the concentration of IAA in the phycosphere changes in natural environments during algal blooms. Furthermore, high concentrations of IAA can have algicidal effects, leading to speculation that IAA accumulation may also be one of the triggers during the extinction stage of algal blooms. Although the involvement of IAA in algal–bacterial interactions as a signaling molecule has been partially verified in co-culture experiments, further analysis of auxin reactions in algal models is needed. Additionally, there needs to be a focus on identifying the specific molecular mechanisms of IAA action. With the advent of new omics techniques, and associated generation of data at the genomic, transcriptomic, proteomic, and metabolic levels, our understanding of the function of IAA in interactions between hosts and microbes in cross-kingdom cell-to-cell communication will be greatly enhanced.

## Author contributions

XC and XL: conceptualization and writing the manuscript. ZC and LC: project administration. JZ, MMT, and JW: review, editing, and revising. All authors contributed to the article and approved the submitted version.
